# Feasible strategies for studying the involvement of DNA methylation and histone acetylation in the stress-induced formation of quality-related metabolites in tea (*Camellia sinensis*)

**DOI:** 10.1038/s41438-021-00679-9

**Published:** 2021-12-01

**Authors:** Jie Yang, Dachuan Gu, Shuhua Wu, Xiaochen Zhou, Jiaming Chen, Yinyin Liao, Lanting Zeng, Ziyin Yang

**Affiliations:** 1 Key Laboratory of South China Agricultural Plant Molecular Analysis and Genetic Improvement & Guangdong Provincial Key Laboratory of Applied Botany, South China Botanical Garden, Chinese Academy of Sciences, No. 723 Xingke Road, Tianhe District, Guangzhou, 510650 China; 2 University of Chinese Academy of Sciences, No. 19A Yuquan Road, Beijing, 100049 China; 3 Center of Economic Botany, Core Botanical Gardens, Chinese Academy of Sciences, No. 723 Xingke Road, Tianhe District, Guangzhou, 510650 China

**Keywords:** Plant stress responses, Secondary metabolism

## Abstract

Tea plants are subjected to multiple stresses during growth, development, and postharvest processing, which affects levels of secondary metabolites in leaves and influences tea functional properties and quality. Most studies on secondary metabolism in tea have focused on gene, protein, and metabolite levels, whereas upstream regulatory mechanisms remain unclear. In this review, we exemplify DNA methylation and histone acetylation, summarize the important regulatory effects that epigenetic modifications have on plant secondary metabolism, and discuss feasible research strategies to elucidate the underlying specific epigenetic mechanisms of secondary metabolism regulation in tea. This information will help researchers investigate the epigenetic regulation of secondary metabolism in tea, providing key epigenetic data that can be used for future tea genetic breeding.

## Introduction

Tea (*Camellia sinensis*) is an important economic crop in China. Indeed, tea plants have been cultivated and used in China for thousands of years. Tea is currently second only to water as the most important beverage worldwide^1^. Compared with other plants, tea contains unique secondary metabolites, including catechins, amino acids (mainly L-theanine), caffeine, and aroma compounds, giving it unique color, aroma, and flavor qualities while also influencing human health^2,3^. Hence, there has been increasing interest in research on the formation of compounds related to tea function and quality. These compounds are mainly formed during growth (i.e., the preharvest stage) and the processing (i.e., the postharvest stage)^4–6^. Previous studies have explored the formation of function- or quality-related metabolites in enzymatic and nonenzymatic (i.e., chemical) reactions^1,4^, and the formation of tea metabolites from a plant biology perspective has also been investigated. Thus, secondary metabolism and associated regulatory processes in tea plants are important research topics.

Environmental stresses affect the formation of secondary metabolites in plants *via* physiological and biochemical mechanisms in adaptation to environmental conditions and resistance to adverse effects caused by external stresses^7^. Most of the traditional theories regarding plant metabolic responses to stress are based on analyses of plant stress tolerance. In general, tea is exposed to multiple stresses during the preharvest and postharvest stages of production, which can induce enrichment of secondary metabolites closely related to quality or function^3,4^. For example, shading is commonly used in the preharvest stage to change the light conditions to increase the contents of flavor-related compounds, including amino acids and aromatics, while decreasing the abundance of polyphenols such as catechins (reducing bitterness)^5^. Therefore, a thorough characterization of the mechanism by which these stressors induce the formation of secondary metabolites in tea and the application of the mechanism to improve the quality of tea components may lead to the development of new methods for safely enhancing tea quality.

**Fig. 1:**
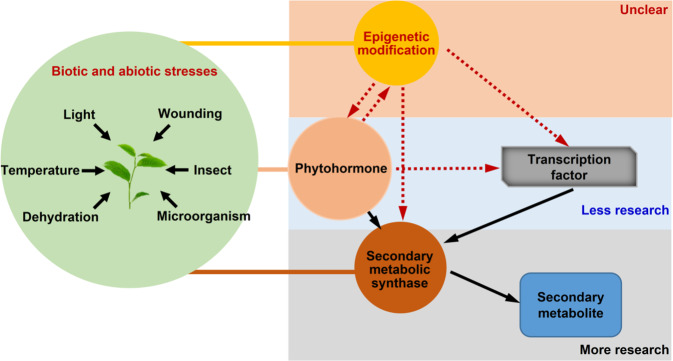
Study on secondary metabolism of tea plants under stress response. As tea is a nonmodel plant species that lacks a well-established genetic transformation system, there has been relatively little epigenetic-related research on this plant. Thus, researchers are faced with many challenges when conducting epigenetic studies on the regulation of secondary metabolism in tea. For example, it is unclear how to verify whether epigenetics is involved in the regulation of secondary metabolism in tea and what the main epigenetic factors involved in the regulation are. In this review, we explore research strategies for investigating the epigenetic control of secondary metabolism in tea on the basis of the genetic characteristics of these plants, focusing on the following two major epigenetic modifications as examples: DNA methylation and histone deacetylation. First, we review epigenetic regulation of plant secondary metabolism and explore the possibility that DNA methylation and histone acetylation are involved in the upstream regulation of key genes of tea quality-related metabolites in tea induced by stress. Second, possible regulatory patterns of DNA methylation and histone acetylation are summarized. Third, a feasible strategy for studying epigenetic regulation of plant secondary metabolism in tea is proposed. The main objective of this review is to summarize research strategies for studying the epigenetic regulation of tea secondary metabolism and providing key epigenetic factors that can be used for future tea genetic breeding.

To date, studies on the mechanism underlying stress-induced secondary metabolites in tea have mainly focused on associated genes, proteins, and metabolites, especially the stress-induced increase in target metabolite contents, which is mainly due to upregulated expression of key synthesis-related genes. For example, exposure to low temperatures and continuous wounding synergistically increase indole, jasmine lactone, and (*E*)-nerolidol contents in oolong tea mainly because of stress-induced expression of *CsTSB2*, *CsLOX1*, and *CsNES*, which are key genes mediating the synthesis of these aroma compounds^8–12^. However, regarding the upstream regulatory mechanisms, there are only a few reports describing the involvement of some plant hormones and transcription factors. Specifically, upstream regulation of the formation of tea aroma compounds synergistically induced by low temperatures and continuous wounding is mainly related to jasmonic acid and the transcription factor *CsMYC2*^11^. Other factors involved in upstream regulatory activities, such as epigenetic regulators, remain poorly understood. Epigenetic regulation in various plants is closely related to stress and secondary metabolite production^13–18^, but there is relatively little research with regard to tea^19,20^. Accordingly, there is an urgent need for studies on the epigenetic regulation of tea quality (Fig. 1), as the paucity of research on the upstream signaling mechanisms of secondary metabolism has not only limited molecular analyses of tea but has also restricted the development of novel methods for improving its quality.

## DNA methylation and histone acetylation may be the main upstream regulators of the expression of key genes influencing stress-induced tea quality-related metabolism

In plants, DNA methylation is primarily responsible for regulating physiological and biochemical processes such as gene expression, cell differentiation, metabolism, and stress responses^21^. For example, when plants are stimulated by external stresses, the genomic DNA methylation status changes (i.e., increases or decreases) to modulate chromatin structure and expression of genes related to responses to environmental stimuli^22^. Histone modifications, including acetylation, methylation, phosphorylation, and ubiquitination, involve covalent changes to histone amino acids. Histone acetylation relaxes nucleosome structures, making DNA more receptive to transcription factors that activate specific genes; in contrast, histone deacetylation and partially methylated histone sites (e.g., K9 and K27) tighten chromatin and inhibit transcription^23,24^. Histone acetylation is closely related to gene silencing, seed germination, morphogenesis, and stress responses^25^, and studies have confirmed that many types of epigenetic changes regulate various plant growth and developmental processes, including flowering, root/stem cell maintenance, hypocotyl elongation, embryogenesis, seed germination, and responses to biotic and abiotic stresses^26,27^. However, in tea plants, epigenetic regulation of plant secondary metabolite formation has not been thoroughly studied. Here, we focus on the role of DNA methylation and histone acetylation modifications on the regulation of secondary metabolites in tea under stress conditions and propose a feasible research strategy.

Compared with epigenetic studies of model plants, progress in nonmodel plants is relatively slow, especially because most nonmodel plants exhibit characteristics of a long growth cycle and abundant population, which determine the uniqueness and innovation of their epigenetic research. With the development of epigenetic research methods over the past two decades, significant progress has been made in research on epigenetic regulation of secondary metabolism, which has improved our understanding of epigenetic regulation of secondary metabolism in nonmodel plants. In recent years, epigenetic modifications have been found to be involved in secondary metabolism regulation in many plants (Table 1). Epigenetic involvement is determined by detecting metabolite contents and gene expression changes after inhibitor treatment^28–30^. Restriction enzyme digestion and bisulfite sequencing are also used to analyze methylation level changes of target genes and the specific mechanism of epigenetic modification regulating secondary metabolism^13,14,16–18,31,32^. Among these studies, DNA methylation and histone acetylation modification are particularly notable.

**Table 1:** Secondary metabolites and epigenetic regulation in plants.

Species	Metabolites	Epigenetic modification	Research methods	References
*Vitis amurensis*	Resveratrol	DNA methylation	5-azaC treatment	^28^
*Populus*	Anthocyanin	Histone methylation/DNA methylation	BS-seq	^29^
*Pyrus*	Anthocyanin	DNA methylation	BSP/McrBC-PCR	^16,18^
*Malus domestica*	Anthocyanin	DNA methylation	BSP/McrBC-PCR	^14,17,18^
*Malus* crabapple	Anthocyanin	Histone acetylation	TSA treatment/BS-seq	^31^
*Solanum lycopersicum*	Carotenoid	DNA methylation	BSP/McrBC-PCR	^13^
*Solanum lycopersicum*	Vitamin E	DNA methylation	BSP/McrBC-PCR	^32^
*Dendrobium nobile* Lindl	Polysaccharide, alkaloid, carotene	DNA methylation	5-azaC treatment	^30^

*5-azaC* 5-azacytidine, *BS-seq* bisulfite sequencing, *BSP* bisulfite sequencing PCR, *TSA* trichostatin A.

As a typical nonmodel plant, tea plants are rich in secondary metabolites. Therefore, it is important to investigate the regulatory role of epigenetic modification in secondary metabolite formation in tea plants. Despite relatively recent molecular investigations in tea, researchers have clarified the effects of epigenetic modifications on specific aspects (Table 2). To elucidate the regulatory role of epigenetics in different aspects of tea plants, models have been selected according to research objectives. For example, from the perspective of genome-wide and overall methylation levels, it was found that DNA methylation and histone acetylation are involved in the evolution and stress response of tea plants, revealing the genetic basis and providing new insight into the mechanism of flavor substance formation and quality regulation of tea^33–37^. From the perspective of transcriptomics, results of analysis of the biological information of epigenetic modification and epigenetic regulatory factors in tea plants have suggested that DNA methylation plays an important role in the regulation of stress response and growth and development^38,39^. From the perspective of proteomics, elucidating the regulation of specific modification sites with regard to the formation of secondary metabolites has provided important insight into the regulatory role of lysine acetylation in secondary metabolism in tea plants^40,41^. Based on changes in the DNA methylation level and histone modification of the target genes, the regulatory factors involved in the regulation of tea processing, low temperature and long illumination have been identified, clarifying the specific regulatory mechanism of the formation of secondary metabolites^19,20,42–44^. These results suggest that epigenetic modifications help to regulate stress responses and secondary metabolite biosynthesis in tea. Accordingly, the stress-induced formation of secondary metabolites in tea may be related to the regulation of DNA methylation and histone acetylation.

**Table 2:** Epigenetic regulation in tea plants.

Epigenetic modification	Research methods	Main research contents	References
Lysine acetylation	Proteome	Nitrogen absorption/assimilation	^40^
Lysine acetylation	Proteome/acetyl-proteome	Leaf color	^41^
Histone deacetylation	Genome-wide/ChIP-qPCR	Proteins/functional characterization	^35^
DNA methylation	MSAP/HPLC	cold acclimation	^36^
DNA methylation	Transcriptional analysis	CsDRM2	^42^
DNA methylation	WGBS	Flowering	^44^
DNA methylation	HPLC/BSP	Anthocyanin	^43^
DNA methylation	HPLC	Growth/development	^33^
DNA methylation	BSP	Transposon silencing/genome size expansion	^34^
DNA methylation	Genome-wide investigation/transcriptional analysis	DNA Methyltransferase/DNA demethylase	^38^
DNA methylation	Genome-wide investigation/expression analysis	DNA demethylase	^39^
DNA methylation	WGBS	Duplicated gene evolution/chilling response	^37^
Histone deacetylation/DNA methylation	ChIP-qPCR	ABA	^19^
DNA methylation	ChIP-qPCR	Indole	^20^

*ChIP* chromatin immunoprecipitation, *MSAP* methylation-sensitive amplification polymorphism, *HPLC* high-performance liquid chromatography, *WGBS* whole-genome bisulfite sequencing, *BSP* bisulfite sequencing PCR.

## Studies on the regulation of DNA methylation and histone acetylation of key genes mediating stress-induced tea quality-related metabolism

Many studies have demonstrated that epigenetic modifications influence plant stress responses, providing new data and directions for epigenetic research^45,46^. Subsequent studies have found a correlation between epigenetic modifications and secondary metabolite contents and quality^47^. In tea, some secondary metabolites are formed in the postharvest stage; this is in contrast with the preharvest stage, during which plants are damaged by various stresses^1^. Secondary metabolites are closely associated with tea quality. Hence, studying the epigenetic status of tea plants under different stress conditions, resolving the epigenetic modifications induced by stresses, and revealing key factors influencing tea quality will provide the basis for future investigations of the molecular mechanisms underlying processes affecting tea quality.

**Fig. 2:**
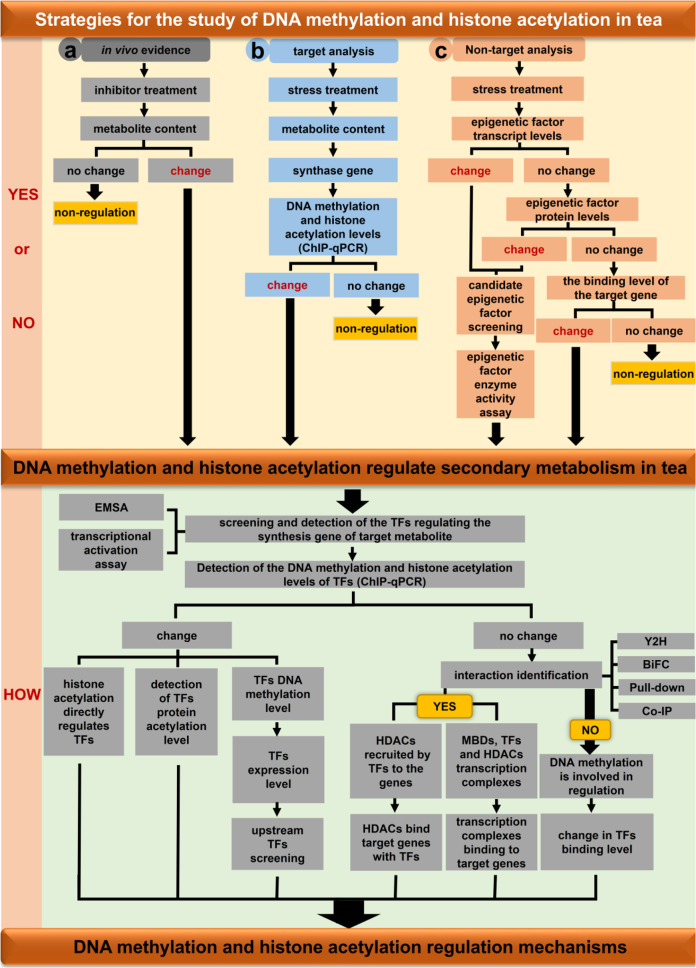
Strategies for studying epigenetic modifications under stress in tea plants. **a** In vivo evidence of inhibitor treatment. **b** Analysis of target metabolites under stress. **c** Analysis of transcription level of epigenetic factors under stress. YES or NO denotes judging whether DNA methylation and histone acetylation are involved in the regulation of secondary metabolism in tea plants. HOW represents the mechanism of DNA methylation and histone acetylation involvement in the regulation of secondary metabolism in tea plants. TFs transcription factors, HDACs histone deacetylases, MBDs methyl-binding domain protein, ChIP chromatin immunoprecipitation, EMSA electrophoretic mobility shift assay, Y2H yeast two-hybrid, BiFC bimolecular fluorescence complementation, Co-IP coimmunoprecipitation.

Nevertheless, epigenetic-based research on tea lags behind that of model plants because of its long growth cycle, large genomes, and varietal diversity. For example, unlike *Arabidopsis*, it is difficult to obtain tea mutants and a stable genetic transformation system. Accordingly, generating accurate and valid in vivo experimental data when functionally characterizing target genes is challenging, which hinders exploration of the consequences of epigenetic modifications on secondary metabolites in tea. Furthermore, epigenetic regulation in tea exposed to abiotic stress is a dynamic process that requires sophisticated experimental techniques for analysis [e.g., polyphenols may affect reverse crosslinking during chromatin immunoprecipitation (ChIP) experiments or cause DNA damage^48^]. Thus, analyzing the epigenetic mechanism in tea under stress conditions is very difficult. In general, determining whether DNA methylation and histone deacetylation are involved in the regulation of secondary metabolism under these conditions is critical and should be the first step in the study of stress-induced epigenetic modifications in tea. This can be achieved in the following three ways (Fig. 2). (1) Samples are treated with DNA methylation and histone acetylation inhibitors, after which the secondary metabolite contents in control and treatment groups are analyzed to obtain direct evidence of a correlation in vivo. If there is no difference in secondary metabolite contents between the groups, DNA methylation and histone acetylation are not involved in regulating the secondary metabolism related to this process. In contrast, significant differences in secondary metabolite contents between the groups suggest that DNA methylation and histone acetylation help to regulate the secondary metabolism related to this process. (2) Secondary metabolite contents in tea are determined under diverse stress conditions^49^, and levels of DNA methylation and histone acetylation of the synthase genes involved in the regulation of secondary metabolites are analyzed. (3) Transcription of DNA methyltransferase, DNA demethylase, histone acetyltransferase (HAT), and histone deacetylase (HDAC) genes in tea are quantified under various stress conditions, and the data are analyzed *via* nontargeted correlation analyses. Significant differences in the expression of genes related to DNA methylation and histone acetylation under control and stress conditions suggest that both are involved in the secondary metabolism that regulates this process. If there is no significant difference between sample groups, differences in the abundance of the encoded DNA methylation-related and histone acetylation-related enzymes are determined, and significant differences in enzyme levels between control and stress conditions indicate that DNA methylation and histone acetylation are involved in the secondary metabolism that regulates this process. If there is no significant difference, the binding of HDACs to target genes is evaluated. If the ability of HDACs to bind to the target genes varies between control and stress conditions, histone acetylation is involved in regulating the secondary metabolism of this process, whereas a lack of a significant difference indicates a lack of involvement.

In the first determination method (Fig. 2, a route), diverse DNA methylation and histone acetylation inhibitors are available for analyses (Table 3). Regarding DNA methylation, tea leaves can be treated with DNA methyltransferase inhibitors to investigate secondary metabolite contents and expression of related genes and to verify whether DNA methylation is important for regulating secondary metabolite biosynthesis. Common DNA methyltransferase inhibitors used for plant studies are 5-azacytidine (5-azaC), 5-aza-2′-deoxycytidine (5-aza-dC), and zebularine, of which 5-azaC is still the most widely used. In terms of histone deacetylation, HDAC inhibitors are divided into four categories on the basis of structural features. Hydroxamic acid salts, such as trichostatin A (TSA) and diallyl disulfide, are natural HDAC inhibitors^50,51^. Fatty acids, such as sodium butyrate, competitively bind to the zinc region of RPD3 and HD2-type HDACs to inhibit activity^52^. Cyclic peptides, including the HC toxin, have a high affinity and specificity for HDACs^53,54^. Benzamides include sirtinol, which is a specific inhibitor of SIRT1 and SIRT2^55^. Nicotinamide noncompetitively inhibits HDAC activity through sirtuins^56^. Among these inhibitors, TSA was the first HDAC inhibitor identified, and it is often used in botanical studies. Specifically, TSA enhances histone acetylation and is useful for studying the regulation of gene expression via acetylation modifications. In addition, TSA decreases genomic 5-methylcytosine levels, but the specific mechanism remains unknown^57^. Thus, DNA methylation and HDAC inhibitor treatments can detect changes in DNA methylation and histone acetylation levels. In addition, changes in related gene expression and secondary metabolite contents can be analyzed to quickly verify the regulatory effects, if any, of DNA methylation and histone acetylation modifications. Regardless, inhibitor treatments cannot elucidate the specific mechanisms underlying DNA methylation and histone acetylation. Moreover, they cannot be employed to identify precise DNA methyltransferases, DNA demethylases, HATs, or HDACs for analysis of the regulatory mechanisms involved. Inhibitor treatments are also relevant for verifying the regulatory effects of DNA methylation and histone acetylation in tea. However, because the genetic background of tea is still unclear and most of the relevant genomic databases are not publicly available, specific DNA methylation and histone acetylation loci in tea cannot be verified by inhibitor treatments. Nevertheless, inhibitor treatment results indicate that DNA methylation and histone acetylation are potentially useful for enhancing the economic value of tea; they may also lead to the development of new strategies for unraveling functional metabolism in tea plants.

**Table 3:** DNA methylation inhibitors and histone deacetylase inhibitors in plant studies.

Species	Inhibitor	Epigenetic	References
*Daucus carota* L.cv*. Koushingosun*	5-azaC	DNA methylation	^98^
*Arabidopsis*	5-azaC	DNA methylation	^99^
*Solanum ruiz-lealii*	5-azaC	DNA methylation	^100^
*Solanum tuberosum*	5-azaC	DNA methylation	^101^
*Arabidopsis*	5-aza-dC	DNA methylation	^52^
*Arabidopsis*	Zebularine	DNA methylation	^99^
*Arabidopsis*	TSA	RPD3 and HD2-type histone deacetylase	^50^
*Brassica napus*	Sodium butyrate	RPD3 and HD2-type histone deacetylase	^102^
*Arabidopsis*	Sirtinol	SIR-type histone deacetylase	^55^
*Arabidopsis*	Nicotinamide	SIR-type histone deacetylase	^56^
*Arabidopsis*	Diallyl disulfide	SIR-type Histone deacetylase	^51^
*Pisum sativum* L. Cv. Alaska 2B	HC toxin	Histone deacetylase	^53^

*5-azaC* 5-azacytidine, *5-aza-dC* 5-aza-2′-deoxycytidine, TSA trichostatin A.

According to available studies, there are various techniques for epigenetic research on secondary metabolites, such as genomics, proteomics, transcriptomics, and analysis of specific modification sites^58^. However, it is difficult to decide which research method to implement. Here, we use DNA methylation and histone acetylation as examples to clarify common experimental techniques for detecting DNA methylation and histone modification levels and discuss their advantages and disadvantages (Table 4).

**Table 4:** Techniques for detecting DNA methylation and histone acetylation.

Epigenetic modification	Experimental techniques	Techniques principle	Advantage	Disadvantages
DNA methylation	MSAP	Enzyme digestion	Easy operation; wide coverage	Only recognizes the CCGG site
	McrBC-PCR	Enzyme digestion	Easy operating; less dosage of DNA	Cutting site uncertain; cutting site easily overlaps
	HPLC	Hydrolysis treatment, UV detection	Whole genome; high sensitivity; high throughput; high resolution	Results inaccurate; hardware expensive; difficult operation
	BS-seq	Bisulfite treatment	High accuracy; quantification	Process complex; time-consuming and expensive
	WGBS	Bisulfite treatment	Wide range; low cost; high efficiency; high accuracy	Large amount of data; analysis of difficulties
	MeDIP-qPCR	Antibody enrichment	Sensitively; rapidly; accurately quantifies	Sample purity required high
	MeDIP-seq	Antibody enrichment	High specificity; high accuracy; good repeatability; high sensitivity	High specificity of antibody; high cost of sequencing
	PacBio	Depends on DNA polymerase activity	No sulfite treatment; no restriction sites; long read long sequencing; high accuracy	Easy to make mistakes; high cost of sequencing
	Nanopore	Electrical signal detection	Better raw data quality; relatively easy to use; low price	Signal instability; poor accuracy; high error rate
Histone modification	ChIP-qPCR	Antibody enrichment	Narrow application range; accurate information	Sample purity required high
	ChIP-seq	Antibody enrichment	Wide range; large amount of information	Sample quantity large; data quantity large; easy to appear false-positive

*ChIP* chromatin immunoprecipitation, *MSAP* methylation-sensitive amplification polymorphism, *HPLC* high-performance liquid chromatography, *WGBS* whole-genome bisulfite sequencing, *BSP* bisulfite sequencing PCR, *MeDIP* methylated DNA immunoprecipitation

Many DNA methylation detection techniques have been developed in recent years. These methods can be divided into the following three categories based on the sample DNA preprocessing involved: (1) pretreatment with restriction enzymes^59^, which mainly includes methylation-sensitive amplification polymorphisms (MSAPs)^60^ and McrBC^61,62^; (2) pretreatment with bisulfite treatment^63^, including high-performance liquid chromatography (HPLC), bisulfite sequencing PCR (BSP)^63,64^, whole-genome bisulfite sequencing (WGBS)^65^, third-generation sequencing methods single-molecule real-time (SMRT) sequencing by PacBio^66^ and Oxford nanopore sequencing^67,68^; and (3) preconditioning *via* affinity enrichment^69^, such as methylated DNA immunoprecipitation (MeDIP) and MeDIP coupled with sequencing (MeDIP-seq)^70,71^. DNA methylation detection techniques need to be selected according to the specific research objectives, availability of reference genome sequences, and sample size. There are few available methods for studying histone modifications, with ChIP-based methods being the most common^72^. ChIP-based techniques involve cell fixation, chromatin fragmentation, ChIP, reverse crosslinking, DNA purification, and DNA identification^73^. ChIP can detect dynamic interaction between *trans*-factors and DNA in vivo, and it can also be used to study the relationship between various covalent modifications of histones and gene expression. Furthermore, combining ChIP with other methods broadens its utility. For example, ChIP coupled with qPCR (ChIP-qPCR) and ChIP coupled with next-generation short sequence sequencing (ChIP-seq) are currently the most commonly employed methods for detecting histone modifications.

With the combination of biology, physics, and chemistry technology, DNA methylation and histone acetylation detection technologies will continue to improve, and the combination of multiple sequencing technologies can greatly promote the excavation and analysis of epigenetic studies of secondary metabolites in nonmodel plants and accelerate research on the genetic regulation mechanism of DNA methylation and histone acetylation.

## Coregulation patterns of DNA methylation and histone acetylation with transcription factors

Many studies have shown that epigenetic regulation is involved in the response to abiotic stress in plants, and this new understanding has resulted in new data and directions for epigenetic research. The epigenetic regulation of secondary metabolism is a complex process, and more studies are needed to explain the mechanism of its establishment and maintenance as well as the relationship between various regulatory factors. Therefore, it is very difficult to study the specific mechanism. The existence of transcription factors greatly solves this problem. Transcription factors generally consist of the following four functional regions: a DNA-binding domain, a transcriptional regulatory region, oligomerization sites, and a nuclear localization signal^74^. The DNA-binding domain determines the specificity of the binding to *cis*-elements, whereas the transcriptional regulatory region activates or represses gene expression. A series of transcription factors recently isolated from higher plants were confirmed to regulate the expression of genes responsive to drought, salinity, low temperatures, hormones, pathogens, and wounding. We summarizes the regulatory patterns by which DNA methylation, histone acetylation, and transcription factors may be involved in the coregulation of secondary metabolism in tea, providing methodological references for future studies.

**Fig. 3:**
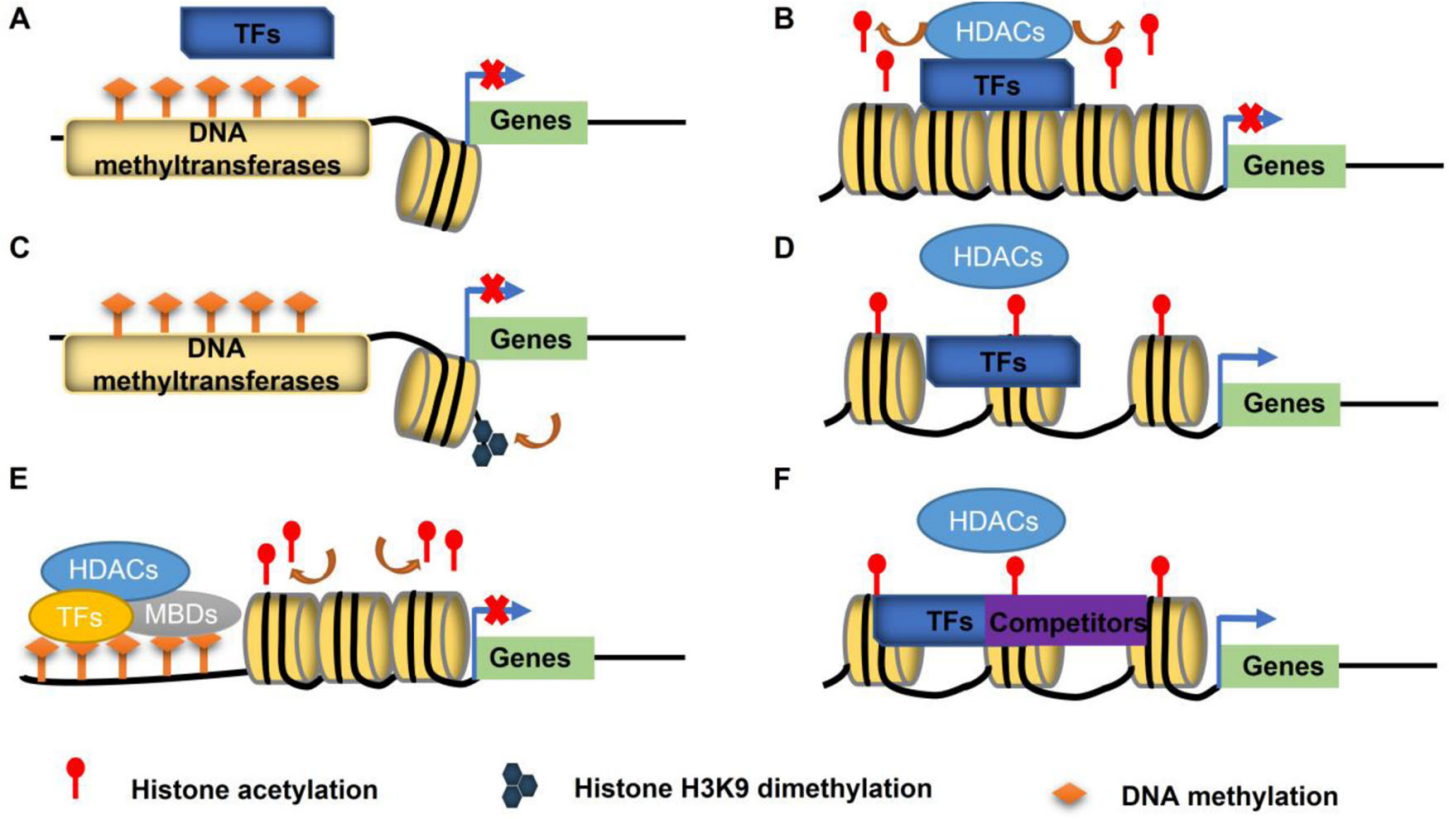
Regulatory patterns of DNA methylation and histone acetylation. **A** DNA methylation inhibits binding by transcription factors. **B** HDACs recruited by and interact with transcription factors to bind target genes. **C** Transcriptional regulation of histone modifications (such as H3K9me2 or histone acetylation). **D** HDACs disengage in interactions with transcription factors to promote the expression of genes. **E** Methyl-binding proteins (MBDs) bind to methylation sites and interact with other transcriptional repressors to form transcriptional repressor protein complexes to inhibit transcription. **F** Competitors interact with transcription factors and relieve the inhibitory ability of HDACs. *TFs* transcription factors, *HDACs* histone deacetylases, *MBDs* methyl-binding domain protein.

Studies have demonstrated that DNA methylation and histone deacetylation are closely related to the chromatin state, higher-order chromosomal structure, gene transcription, signal transduction, and secondary metabolism^21,62^. A methylated gene promoter region affects transcription in three ways. Specifically, methylation inhibits the binding of transcriptional activators or promotes that of transcriptional repressors to directly regulate transcription (Fig. 3A)^75^. In addition, promoting histone modifications that repress transcription (e.g., H3K9me2) and inhibiting histone modifications that enhance transcription (e.g., acetylation) indirectly inhibit transcription (Fig. 3C). Furthermore, methyl-binding proteins (MBDs) bind to methylation sites and interact with other transcriptional repressors to form transcriptional repressor protein complexes (Fig. 3E)^76–79^. Histone deacetylation regulates transcription by altering histone acetylation levels of target genes, and they are primarily recruited by and interact with transcription factors (Fig. 3B). The inhibitory effect can be reversed in two ways, resulting in increased histone acetylation levels. First, HDACs disengage from transcription factors targeting specific genes (Fig. 3D). Second, other proteins interact with transcription factors and compete with HDACs to bind to a target gene, thereby decreasing the inhibitory effect of HDACs (Fig. 3F)^80–83^.

The specific mechanisms by which epigenetic modifications (i.e., DNA methylation and histone acetylation) and transcription factors coregulate tea secondary metabolism have been investigated based on the regulatory effects of DNA methylation and histone acetylation on tea secondary metabolism as well as the regulatory patterns of DNA methylation and histone acetylation, which revealed that DNA methyltransferases and HDACs have modifying roles (Fig. 2). DNA methyltransferases and HDACs were first identified as modifying enzymes, after which enzyme activity assays and subcellular localization experiments were performed to clarify their functions and regional specificity, providing the basis for research regarding specific regulatory mechanisms. Elucidation of specific regulatory mechanisms as well as the screening and identification of transcription factors are also important. The electrophoretic mobility shift assay (EMSA) can be used to identify transcription factors binding to genes involved in secondary metabolite synthesis. Moreover, transient transcriptional activation assays are useful for examining the regulatory effects of transcription factors on the expression of these genes. DNA methylation and histone acetylation levels of transcription factor genes have also been determined. Differences in histone acetylation levels of transcription factor genes between control and stress conditions imply that histone deacetylation can affect secondary metabolite contents by regulating the histone acetylation of transcription factor genes during secondary metabolism. Differences in the DNA methylation levels of transcription factor genes between control and stress conditions indicate that the methylation status of transcription factor genes affects expression or modulates the binding of upstream transcription factors. If there is no difference in the DNA methylation and histone acetylation levels of transcription factor genes under control and stress conditions, another regulatory mechanism involving other transcription factors and DNA methylation or histone acetylation is likely involved. Furthermore, genes encoding the identified transcription factors, DNA methyltransferases, DNA demethylases, HATs, and HDACs have been cloned, and monoclonal antibodies have been prepared for corresponding in vivo experiments. To further clarify the specific regulatory mechanisms, interactions between transcription factors and DNA methyltransferases, DNA demethylases, HATs, or HDACs have been evaluated by yeast two-hybrid (Y2H), pull-down, bimolecular fluorescence complementation (BiFC), and coimmunoprecipitation (Co-IP) assays. A lack of interactions suggests that transcription are directly regulated by DNA methylation. Methylation of genes mediating secondary metabolite synthesis inhibits transcriptional activators or promotes the binding of transcriptional repressors, which can be analyzed by ChIP-qPCR experiments comparing the binding of transcription factors to secondary metabolite synthesis genes in response to diverse treatments. However, confirmed interactions lead to two possibilities. First, histone acetylation may have important regulatory functions. More specifically, transcription factors recruit HDACs to secondary metabolite synthesis genes and affect the secondary metabolite contents by regulating the acetylation of these genes to control expression levels (Fig. 3B). Second, secondary metabolite synthesis genes are highly methylated. In addition, methyl-binding proteins bind to the DNA sequence and form a transcriptional repressor complex with transcription factors and HATs or HDACs to regulate expression of the secondary metabolite synthesis genes and modulate secondary metabolite contents (Fig. 3E). Finally, transcription factors and DNA methyltransferases, DNA demethylases, HATs, and HDACs can be used in transient transcriptional activation assays to assess their combined effects on the transcriptional regulation of secondary metabolite synthesis genes and to elucidate the underlying mechanisms (Fig. 2). The continual refinement of experimental techniques will allow researchers to conduct transient transcriptional activation assays using tea protoplasts. Compared with transient gene expression in tobacco or *Arabidopsis* protoplasts, assays involving tea protoplasts eliminate the influence of exogenous genes on the transcription of target genes, resulting in more accurate in vivo data.

It is worth mentioning that there are various modes of epigenetic regulation, and modifying the effects of phosphorylation, ubiquitination, and SUMOylation on epigenetic factors at the protein level has been investigated. For example, in *Arabidopsis*, siz1-mediated ROS1 SUMOylation enhances the stability of ROS1 and positively regulates active DNA demethylation^84^. To provide more clarity on research methods for epigenetic and transcription factors in tea plant, the aspects of protein modification of epigenetic factors are not explored here.

## Perspectives

Recent epigenetic studies have generated a wealth of new information on DNA methylation and histone acetylation modifications. Moreover, methods for analyzing DNA methylation and histone acetylation have improved, enabling more precise examinations of the regulatory effects of these epigenetic changes on plant gene expression and the synthesis of secondary metabolites.

In the future, several key issues need to be considered or addressed when using these approaches in tea. First, the limitation of materials, which is the key problem, needs to be solved. Due to the lack of a stable genetic transformation system, epigenetic modification research is limited in tea. Therefore, it is urgent to develop an efficient genetic transformation system for tea. Second, the selection of regulatory patterns, from the genomic level to the gene level of specific regulatory mechanisms, is a step-by-step process, and it is necessary to design the most appropriate research strategy for each step. Finally, basic research theory is expected to improve the quality and environmental adaptability of tea.

Compared with *Arabidopsis*, it is difficult to obtain direct evidence of epigenetic regulation in tea, and the key is the limitation of research materials. In view of the lack of tea plants without corresponding mutants and stable genetic materials, researchers have continuously explored transient expression in tea plants, and virus-induced gene silencing (VIGS)^85,86^ and antisense oligonucleotide (AsODN) technology comprises a fast and effective method to identify the function of genes in tea and tea epigenetics^87–89^. The latest research reports separation technology of tea protoplasts and localization of biosynthetases of special metabolites, confirming the possibility of transient protoplast expression and providing means for the research on epigenetics in tea^90^.

The recent development and application of various omics-based techniques (e.g., transcriptomics, genomics, and metabolomics) has resulted in significant breakthroughs in research regarding the evolution of tea species, structural variations, metabolite synthesis, and genetic breeding. Previous studies have clarified the evolution of cultivated tea plants and revealed whole-genome duplication events associated with the genetic diversity among tea resources^91–93^, also elucidating the genetic basis of tea flavors and quality as well as ecological adaptations and providing new insight into the formation and quality of tea flavor-related compounds^94,95^. Recent studies have also identified molecular and metabolic markers relevant for tea breeding and functional genomics studies^96,97^. From the perspective of regulatory patterns, the application of multiomics approaches has provided a solid foundation for the study of epigenetic modifications, which will help to clarify the epigenetic regulatory network in tea plants. A more comprehensive analysis may be conducted in conjunction with genomics research methods to identify specific epigenetic regulatory factors and analyze their regulatory mechanism on secondary metabolism in tea plants. It is expected that such research results will be applied for the evaluation and screening of tea plant resources, improvement of tea plant varieties, and targeted regulation of metabolites to enhance the economic value of theoretical research.

## Conclusion

DNA methylation and histone acetylation are important modifications that control gene transcription activation and silencing, and they have important regulatory effects on secondary metabolic regulation in tea, but the specific regulatory mechanism remains to be elucidated. Hence, this review proposes a research strategy involving DNA methylation and histone acetylation to regulate secondary metabolism in tea, including rapid identification of DNA methylation and histone acetylation involved in the regulation of secondary metabolism by inhibitor treatment. Changes in the apparent modification level of metabolite synthase genes can be analyzed to identify whether DNA methylation and histone acetylation are involved in the regulation of secondary metabolism. It is also possible to identify whether DNA methylation and histone acetylation are involved in the regulation of secondary metabolism by analyzing changes in expression of DNA methylation- and histone acetylation-related factors under stress. Various possible research modes of DNA methylation and histone acetylation involved in the regulation of tea secondary metabolism have been proposed, and feasible and effective solutions (VIGS, AsODNs, and protoplast transformation) overcome the limitations of the most critical research materials in research strategies. The correct application of this research strategy will promote the development of studies on epigenetic regulation of tea secondary metabolism and reveal key epigenetic information that can be used for future tea genetic breeding.
